# Therapeutic Targeting of NF-κB in Acute Lung Injury: A Double-Edged Sword

**DOI:** 10.3390/cells11203317

**Published:** 2022-10-21

**Authors:** Michelle Warren Millar, Fabeha Fazal, Arshad Rahman

**Affiliations:** Department of Pediatrics (Neonatology), Lung Biology and Disease Program, University of Rochester School of Medicine and Dentistry, Rochester, NY 14642, USA

**Keywords:** endothelial cells, transcription factors, signal transduction, lung inflammatory injury

## Abstract

Acute lung injury/acute respiratory distress syndrome (ALI/ARDS) is a devastating disease that can be caused by a variety of conditions including pneumonia, sepsis, trauma, and most recently, COVID-19. Although our understanding of the mechanisms of ALI/ARDS pathogenesis and resolution has considerably increased in recent years, the mortality rate remains unacceptably high (~40%), primarily due to the lack of effective therapies for ALI/ARDS. Dysregulated inflammation, as characterized by massive infiltration of polymorphonuclear leukocytes (PMNs) into the airspace and the associated damage of the capillary-alveolar barrier leading to pulmonary edema and hypoxemia, is a major hallmark of ALI/ARDS. Endothelial cells (ECs), the inner lining of blood vessels, are important cellular orchestrators of PMN infiltration in the lung. Nuclear factor-kappa B (NF-κB) plays an essential role in rendering the endothelium permissive for PMN adhesion and transmigration to reach the inflammatory site. Thus, targeting NF-κB in the endothelium provides an attractive approach to mitigate PMN-mediated vascular injury, not only in ALI/ARDS, but in other inflammatory diseases as well in which EC dysfunction is a major pathogenic mechanism. This review discusses the role and regulation of NF-κB in the context of EC inflammation and evaluates the potential and problems of targeting it as a therapy for ALI/ARDS.

## 1. Introduction

Acute lung injury/acute respiratory distress syndrome (ALI/ARDS) is a common cause of respiratory failure in critically ill patients, and is most often caused by pneumonia, sepsis, chemical inhalation, trauma, or ischemia reperfusion injury [1]. ALI/ARDS is frequently a prelude to a wide spectrum of pathologic processes, including multiple organ dysfunction syndrome [2,3], and is associated with a high mortality rate of ~40% [1,3]. Most recently, ALI/ARDS was the major cause of death in coronavirus disease 2019 (COVID-19) patients. It has been reported that ARDS occurred in approximately one-third of hospitalized COVID-19 patients with an unacceptably high mortality rate of ~70% in these (COVID-19/ARDS) cases [4,5]. As of August 2022, severe acute respiratory syndrome coronavirus 2 (SARS-CoV-2) infection has caused ~6.4 million deaths worldwide, of which more than 1 million deaths occurred in the United States alone [6]. Importantly, ~90% nonsurvivors of COVID-19 had ARDS, suggesting that the majority of COVID-19 deaths are attributable to ARDS [4]

ARDS was first defined in 1967 by Ashbaugh et al. [7,8] as a clinical syndrome of noncardiogenic pulmonary edema that presents with progressive arterial hypoxemia, reduced lung compliance, the increased work of breathing, and the need for mechanical ventilation. Since then, considerable diagnostic advancements and mechanistic understanding of the disease progression and resolution have been achieved. However, these advances have not translated into an effective pharmacotherapy for ALI/ARDS, and the current therapeutic options are limited to supportive care [1,3]. The treatment of ALI/ARDS is complicated by its heterogeneity; it can be caused by systemic insults (sepsis or trauma), or by direct inhalation of noxious agents (bacterial or viral infection) [2]. In addition to its high mortality rate, ALI/ARDS survivors have reported long-term pulmonary, cognitive, and muscular deficits [1]. The COVID-19 pandemic has further highlighted the desperate need for effective drugs to control ALI/ARDS.

### 1.1. Pulmonary Endothelium: A Key Orchestrator of Inflammatory Lung Injury

**Figure 1:**
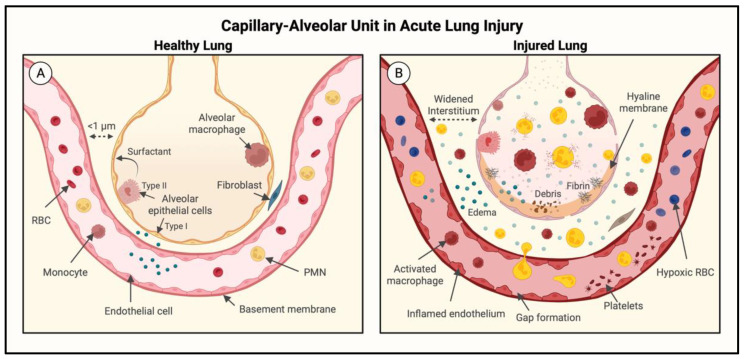
The capillary-alveolar unit in healthy and injured lung. (**A**) In the healthy lung, the intact capillary-alveolar unit (endothelium and epithelium) prevents the influx of immune cells and fluid into the interstitium and alveoli. (**B**) In the injured lung, disruption of the endothelial and epithelial barriers causes pulmonary edema, inflammatory cell recruitment (primarily polymorphonuclear leukocytes (PMNs)), and hypoxemia.

A major pathogenic mechanism of ALI/ARDS is dysregulated inflammation, as characterized by massive infiltration of polymorphonuclear leukocytes (PMNs) into the interstitial and alveolar spaces and the associated damage of the capillary-alveolar barrier, ultimately leading to protein-rich pulmonary edema and the resulting hypoxemia. The pulmonary endothelium is a key structural and functional component of the capillary-alveolar unit. In healthy lung, endothelial cells (ECs) provide an antiadhesive, anticoagulant, and relatively restrictive (semipermeable) barrier that serves to limit the passage of inflammatory cells, maintain blood fluidity, and allow minimal filtration of fluid (Figure 1A). Coupled with an intact alveolar epithelial barrier, the pulmonary endothelium keeps air spaces relatively dry and free of inflammatory cells, thus ensuring proper gas exchange [9,10]. The massive surface area of pulmonary capillary ECs enables the strategically positioned lungs to oxygenate the circulating blood before it enters the systemic circulation [11]. Thus, the functional and structural integrity of the pulmonary endothelium is vital for maintaining the pulmonary and systemic cardiovascular homeostasis.

**Figure 2:**
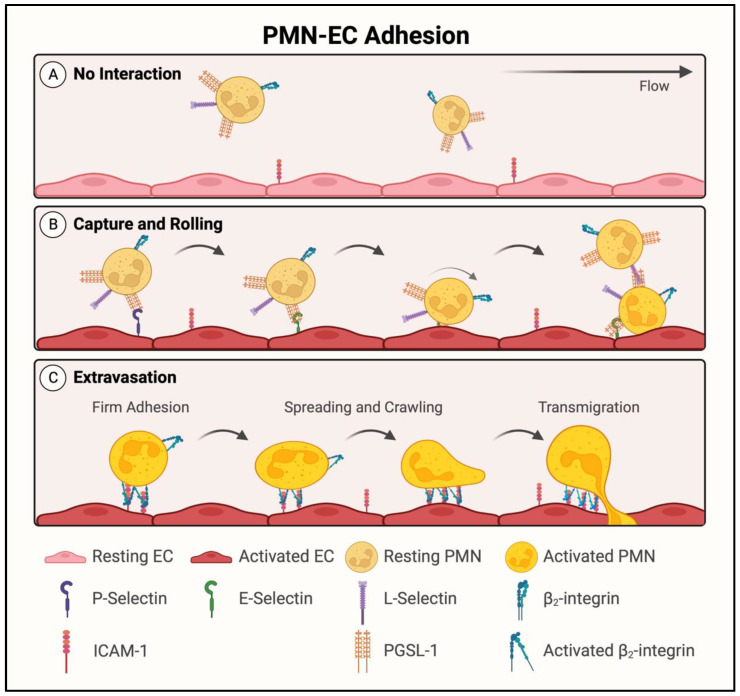
PMN adhesion and transendothelial migration (TEM). (**A**) Resting PMNs express beta-2 (β_2_) integrins and selectin ligands, but unstimulated endothelial cells (ECs) do not express selectins and are, thus, unable to bind circulating PMNs. (**B**) Microbial infections and proinflammatory mediators stimulate the endothelium, and activated ECs express cell surface adhesion molecules (P-selectin, E-selectin, and intercellular adhesion molecule-1 (ICAM-1)), which interact with their counter receptors on PMNs. Primary PMN capture and secondary PMN–PMN tethering are facilitated by interactions between selectins and selectin ligands. As captured PMNs slow in circulation and roll along the endothelium, β_2_ integrins are activated. (**C**) Activated β_2_ integrins interact with EC ICAM-1, which clusters to promote firm EC-PMN adhesion. β_2_-integrin/ICAM-1 interaction facilitates PMN spreading and crawling, and eventually, PMN transendothelial migration.

When lungs are exposed to noxious agents, either by direct inhalation or through pulmonary circulation [3], the endothelium becomes activated following exposure to proinflammatory cytokines (tumor necrosis factor alpha (TNF-α), interleukin-1 beta (IL-1β)) and chemokines (IL-8, macrophage inflammatory protein 2-alpha (MIP2-α)) produced by rapidly responding resident cells, primarily macrophages, in the lung interstitium and alveolus (Figure 1B) [12,13]. These proinflammatory mediators further activate the macrophages and ECs in autocrine and paracrine manners, respectively. The activated endothelium functions as a key orchestrator of inflammation by its ability to become proadhesive for PMNs to adhere and cross the endothelial barrier in order to reach the inflammatory site (Figure 2). The proadhesive phenotype is conferred by the expression of adhesion molecules (P-selectin, E-selectin, intercellular adhesion molecule-1 (ICAM-1)) on the EC surface. These adhesion molecules interact with their counter receptors on PMNs (P-selectin glycoprotein ligand-1 (PSGL-1), beta-2 (β_2_) integrins) in a highly ordered, multistep process that allows ECs to capture circulating PMNs, slow them down to rolling, and eventually, arrest them (Figure 2A,B). This is followed by the spreading, crawling, and positioning of adherent PMNs for their transendothelial migration (TEM) to reach the inflammatory site (Figure 2C). Activation and migration of PMNs by this mechanism in the lung causes PMN alveolitis and the injury of vascular endothelial and alveolar epithelial barriers [14,15,16,17]. In addition to acquiring a proadhesive phenotype, the endothelium is transformed into a prothrombotic surface, causing intravascular coagulation and the generation of thrombin, which in turn, augments these processes by promoting inflammatory and barrier disruptive responses in ECs [18,19,20]. The resulting extravasation of plasma and inflammatory cells leads to pulmonary edema, a reduced alveolar surface area, and impaired gas exchange. Thus, the key players that act in concert to facilitate the adhesion of PMNs to ECs (EC–PMN interaction) for their TEM, increase EC permeability, and promote intravascular coagulation include adhesion molecules (E-selectin, ICAM-1, vascular cell adhesion protein-1 (VCAM-1)); cytokines (TNFα, IL-1β, IL-6); chemokines (IL-8, monocyte chemoattractant protein-1 (MCP-1)), which are upregulated; and anticoagulation proteins (thrombomodulin, endothelial protein C receptor (EPCR)), which are downregulated [21,22]. Importantly, all these proteins are regulated by the transcription factor nuclear factor-kappa B (NF-κB) [21,22]. In this review, we will discuss the role and regulation of NF-κB in the context of EC inflammation and consider the pros and cons of the therapeutic targeting of NF-κB against ALI/ARDS.

### 1.2. NF-κB: A Central Regulator of EC Dysfunction

**Figure 3:**
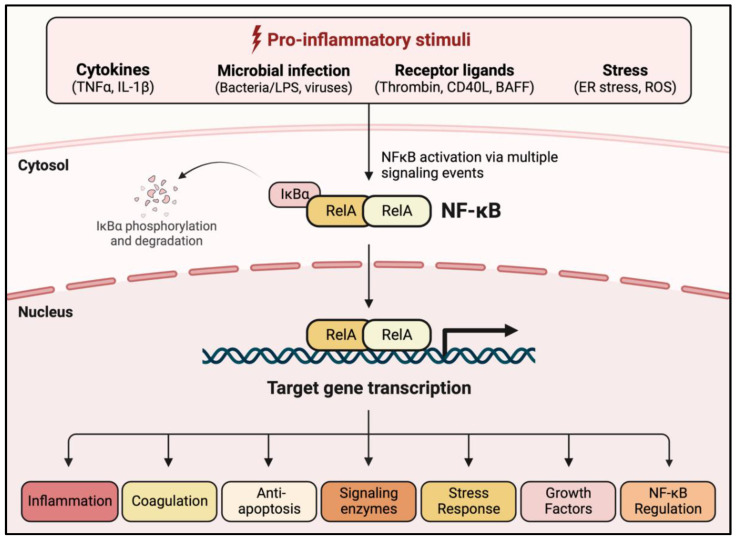
NF-κB activators and cell responses. Nuclear factor-kappa B (NF-κB) is activated by proinflammatory stimuli including cytokines (tumor necrosis factor alpha (TNFα), interleukin-1 beta (IL-1β)), microbial infection (bacteria/lipopolysaccharide (LPS), viruses), receptor ligands (thrombin, CD40 ligand (CD40L), B-cell-activating factor (BAFF)), and stress (endoplasmic reticulum (ER) stress, reactive oxygen species (ROS)). Stimulation liberates the NF-κB dimer secondary to degradation of IκBα (inhibitor of κB) in the cytosol and promotes its nuclear translocation and DNA binding to activate transcription of genes involved in numerous cell responses.

**Figure 4:**
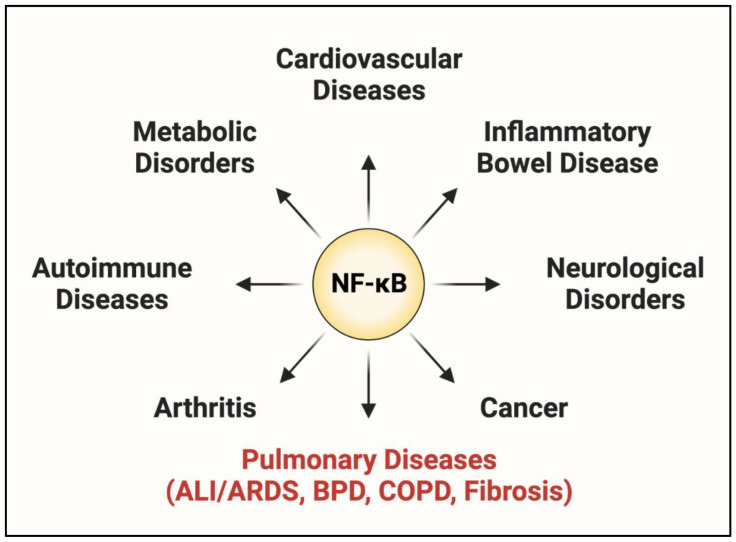
Dysregulated NF-κB signaling is implicated in the progression of numerous disease states.

NF-κB was first identified in 1986 as a nuclear factor binding to a specific conserved sequence in the nuclei of activated B lymphocytes [23]. Since its initial discovery in B cells, NF-κB has been identified in other cell types, including ECs and innate immune cells. NF-κB is considered a master regulator of inflammation because of the essential role it plays in controlling each aspect (evolution and resolution) of the inflammatory response [24,25,26]. Additional functions for NF-κB have been described in cell survival, differentiation, proliferation, stress response, coagulation, tumorigenesis, tissue repair, and its own regulation (Figure 3) [27,28,29]. Aberrant regulation of NF-κB leading to detrimental inflammation is a major driver of many inflammatory diseases including cancer, atherosclerosis, inflammatory bowel disease (IBD), arthritis, and Alzheimer’s disease (Figure 4) [30,31,32]. Lung inflammatory diseases such as ALI/ARDS, asthma, idiopathic pulmonary fibrosis (IPF), bronchoalveolar dysplasia (BPD), and chronic obstructive pulmonary disease (COPD) are also characterized by dysregulated NF-κB activation (Figure 4) [33,34,35,36,37,38]. Of these, ALI/ARDS is particularly driven by EC dysfunction (defined by excessive EC–PMN interaction, increased EC permeability, and activation of intravascular coagulation), which is in turn controlled, in major part, by NF-κB [12,39]. Notably, the severity and lethality of ALI/ARDS caused by pneumonia or sepsis is primarily associated with an NF-κB-mediated “cytokine storm”, in which massive PMN extravasation and the subsequent cytokine release cause rapid deterioration due to widespread inflammation and coagulation [40,41]. More recently, the SARS-CoV-2 spike protein has been shown to activate NF-κB in multiple cell types including ECs and macrophages [42,43]. These findings implicate a similar role of the NF-κB-dependent cytokine storm in COVID-19-associated ALI/ARDS [44].

It has become increasingly clear that the upstream signaling leading to NF-κB activation and the transcriptional output of activated NF-κB that shapes the inflammatory response is influenced by the cellular context and the stimulus involved [19,27]. For example, thrombin and TNFα, which activate the protease-activated receptor 1 (PAR-1, a G-protein-coupled receptor) and TNFα receptor (TNFR), respectively, may engage different signaling events to activate NF-κB to transcribe gene programs that may not be entirely the same. Thus, elucidating the precise signaling events elicited by various inputs to activate NF-κB is of fundamental importance as it has the potential to identify appropriate molecular targets to restrain NF-κB. Given the fundamental role played by ECs in recruiting PMNs to the inflammatory site, not only in the lung, but in other organs as well, they provide an appropriate cellular context for targeting NF-κB to limit the extravasation of PMNs and fluid in the treatment of ALI/ARDS. The following sections discuss the current state of knowledge about NF-κB regulation in the endothelium.

### 1.3. NF-κB Family Members and Inhibitors

**Figure 5:**
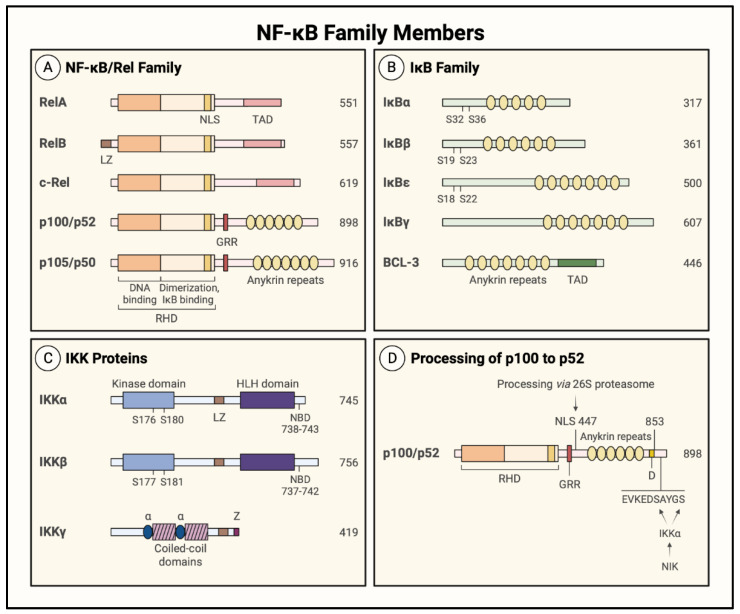
Structure of NF-κB family members. The number of amino acids in each protein is listed on the right. (**A**) The five members of the NF-κB family contain a Rel homology domain (RHD) at their N-terminus. The RHD is comprised of a DNA binding domain, a dimerization/IκB binding domain, and a nuclear localization sequence (NLS). At their C-terminus, the catalytically active RelA, RelB, and c-Rel proteins contain transactivation domains (TADs), whereas p105 and p100 contain a glycine-rich region (GRR) and ankyrin repeats (ovals). The p105 and p100 proteins are catalytically inactive due to the presence of their ankyrin repeats, and the GRR facilitates the cotranslational processing of p105 to p50 and the post-translational processing of p100 to p52. (**B**) The ankyrin repeats (ovals) in IκB proteins interact with the RHD of NF-κB family members and mask its NLS, and serine phosphorylation at the indicated sites promotes their proteasomal degradation. BCL-3 also contains a TAD at its C-terminus, which confers transcriptional activity to p50/BCL-3 and p52/BCL-3 complexes. (**C**) The catalytically active IKKα and IKKβ proteins contain kinase and helix–loop–helix (HLH) domains, and their activity is stimulated by phosphorylation of the kinase domain at the indicated serines. The regulatory protein, IKKγ (or NEMO), contains a zinc finger (Z) domain, two coiled-coil domains, and two a-helical (a) domains, which mediate its association with the NEMO binding domain (NBD) at the C-terminus of IKKα and IKKβ. All three members also contain a leucine zipper (LZ) motif required for protein–protein interaction. (**D**) NF-κB-inducing kinase (NIK) activates IKKα, which phosphorylates the p100 C-terminus to stimulate the post-translational processing of p100 to p52. The p100 protein is cleaved at amino acid 447, and the C-terminus is degraded by the 26S proteasome. The GRR serves as a signal to prevent the proteasome from degrading the N-terminal portion of the protein.

Gene transcription by NF-κB controls vast cellular programs by inducing a wide array of genes. The diverse biological effects of NF-κB raise the question of how distinct signaling activators induce differential gene expression profiles via a single transcription factor. Although the regulatory mechanisms controlling NF-κB are not fully characterized, positive and negative regulatory mechanisms have been identified at multiple levels to tightly control the duration, intensity, and specificity of NF-κB signaling. One aspect of NF-κB biology that leads to specificity in the induced transcriptional programs is the composition of the activated NF-κB dimer. The mammalian NF-κB family is composed of five members: RelA (p65), RelB, c-Rel, p50, and p52, which form 13 described dimer combinations (out of a possible 15) to mediate the transcription of numerous target genes. Each NF-κB member has a highly conserved Rel homology domain (RHD), and a 300-amino acid sequence at their N-terminus containing a nuclear localization signal (NLS) (Figure 5A). The RHD has two immunoglobulin-like subdomains: the N-terminal subdomain binds DNA, and the C-terminal subdomain is responsible for dimerization and binding inhibitory proteins [28,29]. The NF-κB family members fall into two categories depending on their transcriptional competency. RelA (p65), RelB, and c-Rel are synthesized as transcriptionally active proteins owing to the presence of a transactivating domain (TAD), whereas p50 and p52 do not contain TAD and must interact with RelA, RelB, or c-Rel to activate transcription (Figure 5A). The proteins p50 and p52 are also distinct in their synthesis, as they are generated by proteolytic cleavage from precursor proteins p105 and p100, respectively (Figure 5A) [24]. The proteins p105 and p100 are characterized by the presence of a glycine-rich region (GRR) and ankyrin repeats. The presence of GRR serves to control the cotranslational processing of p105 to p50 and post-translational processing of p100 to p52 [27,28,30,31,45]. The p50 and p52 proteins thus generated contain an RHD (dimerization, DNA binding, and IκB binding domains) and GRR (Figure 5A). These features allow the p50 or 52 homodimer and p50/52 heterodimer to act as transcriptional repressors because they are able to bind DNA but lack the TAD necessary for transcriptional activity [46]. However, they can form transcriptionally active heterodimers with RelA or c-Rel. Interestingly, heterodimers of RelB with either p50 or p52 exhibit a greater regulatory flexibility, and function both as an activator and a repressor [27,28,45]. All NF-κB members exist as dimers of a distinct composition, and the prototype of these dimeric forms is the p50/RelA (p50/p65) heterodimer. All NF-κB dimers are present in ECs; however, the p50/p65 heterodimer and p65 (RelA) homodimer are the major drivers of EC inflammation [19,47].

NF-κB proteins contain an NLS to facilitate their translocation to the nucleus, where they bind the promoters of their target genes. However, in resting conditions the inactive NF-κB dimers are sequestered in the cytosol by the interaction with the inhibitor of κB (IκB) proteins that masks the NLS through their interaction with the RHD [27,28,29,30]. The IκB proteins can be grouped into three categories: (1) the typical IκB proteins include IκBα, IκBβ, and IκBε, which undergo signal-induced degradation in the cytoplasm and are resynthesized upon NF-κB activation; (2) the precursor IκB proteins include NF-κB1/p105 and NF-κB2/p100, which undergo processing to remove ankyrin repeats and generate NF-κB family members p50 and p52, respectively; and (3) the atypical IκB proteins are composed of IκBζ, BCL-3, and IκBNS, which are generally inducible and act in the nucleus, unlike the typical and precursor IκB proteins (Figure 5A,B) [27,28,29,45]. The prototypical member of the IκB family is IκBα.

### 1.4. NF-κB Activation Pathways

**Figure 6:**
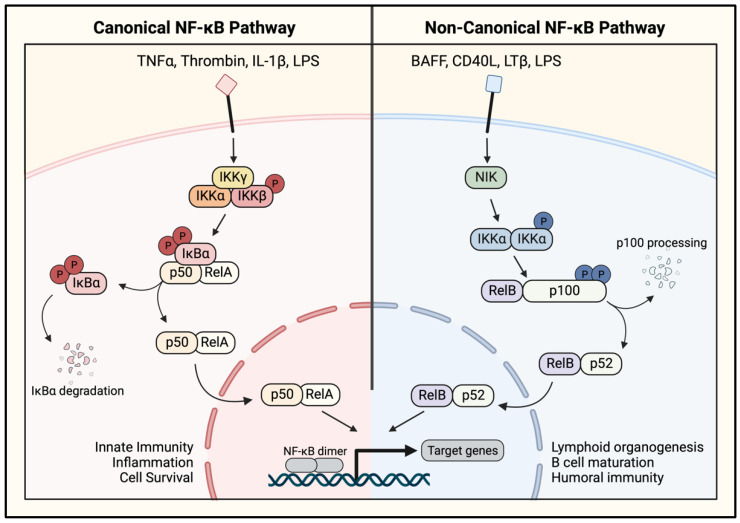
Canonical and noncanonical NF-κB pathways. (**Left**) The canonical pathway is activated by proinflammatory stimuli including TNFα, thrombin, IL-1β, and LPS, among others. Interaction of extracellular stimuli with cell surface receptors results in activation of the IκB kinase (IKK) complex, including the catalytic subunits IKKα and IKKβ. These subunits phosphorylate IκBα, which leads to its ubiquitination and degradation, exposing the NLS on the NF-κB dimer. The NF-κB dimer, predominantly a heterodimer of p50-RelA/p65, translocates to the nucleus and binds DNA to stimulate the transcription of innate immunity, inflammatory, and cell survival genes. (**Right**) The noncanonical pathway is activated by BAFF, lymphotoxin-β (LTβ), CD40L, and LPS, which signal through NF-κB-inducing kinase (NIK) to activate the IKKα homodimer. IKKα phosphorylates the NF-κB protein p100 to stimulate its processing into p52. This processing exposes the NLS on p52, allowing p52/RelB heterodimers to translocate to the nucleus and activate gene transcription. The noncanonical pathway primarily regulates expression of genes involved in B cell maturation and lymphoid organogenesis. (P denotes protein phosphorylation).

Activation of NF-κB is initiated by its release in the cytosol, which is accomplished through the degradation, or in some instances, the dissociation of IκBα [27,28,29,30,45]. IκBα degradation is the classical mechanism of NF-κB release and is contingent upon its phosphorylation at Ser^32^ and Ser^36^ by the IκB kinase complex (IKK) (Figure 6, left). The phosphorylated IκBα undergoes rapid polyubiquitination, which marks it for 26 S proteasome-mediated degradation. This results in the release and unmasking of the NLS in the NF-κB dimer, facilitating the translocation of the freed NF-κB dimer to the nucleus where it activates the transcription of its target genes (Figure 6, left) [27,28,29]. Regulation of the NF-κB pathway by the IκB protein interaction enables rapid signaling because it allows for the formation of latent, preassembled NF-κB dimers that can be readily activated upon proinflammatory stimulation. Inhibition by IκB also represents an inherent negative feedback loop to prevent uncontrolled signaling, because IκBα degradation and NF-κB activation induce the transcription of IκBα [29]. The newly synthesized IκBα interacts with NF-κB dimers to retain them in the cytosol and terminate the transcription.

Activation of IKK represents the convergence point for numerous upstream signaling pathways and is where the distinction first occurs between the canonical pathway (CP) and noncanonical pathway (NP) of NF-κB activation (Figure 6). In the CP, which can be activated by TNFα, IL-1β, lipopolysaccharide (LPS), and thrombin, IκBα is phosphorylated by the IKK complex, comprising two kinase subunits and a regulatory subunit (Figure 5C). The catalytic subunits, IKKα and IKKβ, are tethered by the regulatory subunit, IKKγ (also termed the NF-κB essential modifier (NEMO)) [28,45]. IKKα and IKKβ are activated by phosphorylation of two distinct serine residues, Ser^176^ and Ser^180^ or Ser^177^ and Ser^181^, respectively (Figure 5C). Although both catalytic subunits can phosphorylate IκBα, the action by IKKβ is more predominant [27].

The CP is rapidly activated to respond to an inflammatory stimulus, in contrast with the NP which responds more slowly. The NP can be stimulated by the CD40 ligand, lymphotoxin β (LTβ), and LPS to activate the NF-κB-inducing kinase (NIK) (Figure 6, right). NIK activates the noncanonical IKK complex, comprised only of an IKKα homodimer, which phosphorylates p100 at Ser^176^ and Ser^180^ to induce its processing to the functional p52 protein [24,28] (Figure 5D). The precursor protein p100 acts as an IκB protein in the NP pathway by binding RelB to prevent its nuclear translocation. Proteolytic processing releases the active p52/RelB heterodimer, allowing it to move to the nucleus and activate gene transcription (Figure 6, right). In addition to their distinct signaling mechanisms, the NF-κB pathways differ in their target gene programs. The CP activates the transcription of genes involved in inflammation and injury responses, whereas the NP targets genes involved in lymphoid organogenesis (Figure 6) [19,45].

In addition to its cytosolic activation involving IκBα degradation-dependent release, the activity of NF-κB is tightly regulated in the nucleus as well. The nuclear regulation of NF-κB involves a variety of post-translational modifications which serve to control NF-κB nuclear signaling, as well as its transcriptional output [48,49]. A major controller of NF-κB activity in the nucleus involves the phosphorylation of RelA/p65, which can occur in both the cytoplasm and nucleus. RelA/p65 can be phosphorylated in RHD (Ser^276^ and Ser^311^) or TAD (Ser^529^ and Ser^536^), and this event increases the transactivation potential of RelA/p65 [30]. The phosphorylation site and the kinase involved may vary in a stimulus- and cell-type-specific manner and may contribute to the diversity of induced transcriptional programs. For example, phosphorylation of RelA/p65 at Ser^276^ can be induced by both TNFα and LPS, but via different kinases. Although TNFα engages mitogen- and stress-activated kinase-1 (MSK-1), LPS-induced phosphorylation is mediated by the catalytic subunit of protein kinase A (PKAc). TNFα also promotes Ser^311^ phosphorylation of RelA/p65; however, this event is mediated by a different kinase, protein kinase C zeta (PKCζ). RelA/p65 can also be phosphorylated at Ser^529^ by casein kinase 2 (CK2) or Ser^536^ by upstream NF-κB pathway proteins IKKα and IKKβ [19,50]. The presence of multiple phosphorylation sites and their phosphorylation by various kinases suggest that when phosphorylated in combination, these sites may have cooperative functional effects on the transcriptional activity of RelA/p65 and its output.

RelA/p65 phosphorylation can stimulate additional post-translational modifications to influence its transcriptional potential by recruiting the transcriptional coactivating proteins p300 and cAMP-response element-binding protein (CBP). The recruited p300/CBP or p300/CBP-associated factor (PCAF) induces acetylation of RelA/p65 at multiple sites (Lys^218^, Lys^221^, Lys^310^, and Lys^122/123^) to modulate its transcriptional activity and interaction with DNA or IκB [51]. Acetylation at Lys^218^, Lys^221^, or Lys^310^ each enhances the transcriptional activity of RelA/p65, but by different mechanisms. The acetylation of Lys^218^ prevents the association of RelA/p65 with IκBα, whereas the acetylation of Lys^221^ stabilizes RelA/p65 binding to the κB site; together, these acetylation events prolong the binding of nuclear RelA/p65 to the DNA and increase the transcription of target genes [30,48,51]. In contrast, the acetylation of Lys^310^ increases the transcriptional activity of RelA/p65 without affecting its DNA binding activity or interaction with IκBα [30,48].

## 2. Signaling to NF-κB in the Endothelium

In the majority of cases, signaling triggered by proinflammatory mediators are funneled through IKK to activate NF-κB. Once IKKs are activated, downstream events leading to the nuclear translocation of NF-κB dimers follow (Figure 6). However, the signals transmitted from cell surface receptors to IKK phosphorylation could vary depending upon the proinflammatory stimulus and the cellular context. A distinct set of signaling proteins and adaptor molecules are recruited and activated by a given receptor system to create a specific pathway leading to IKK activation.

In the endothelium, IKK/NF-κB can be activated by a host of stimuli to induce inflammatory responses. Activated NF-κB triggers the expression of target genes that suppress antiadhesive, anticoagulant, and barrier-protective mechanisms and transforms the vasculature into a proadhesive, procoagulant, and leaky barrier that facilitates extravasation of inflammatory cells and fluid into the airspace, impairing the lung function (Figure 1). Consistent with this, the selective blockade of the EC-intrinsic NF-κB pathway reduced lung inflammatory injury and mortality, suppressed coagulation, and protected against vascular dysfunction in mice with sepsis [22,33,52]. The following sections explore signaling mechanisms of endothelial IKK/NF-κB activation in response to proinflammatory stimuli, particularly those induced by thrombin and TNFα. TNFα and thrombin represent an important interface of the inflammation–coagulation axis, and thus, the signaling pathways activated by them may serve to create a vicious cycle where inflammation and coagulation can each amplify the other, resulting in exuberant and persistent inflammation characteristics of ALI, particularly in the setting of sepsis and pneumonia [19,53]. Given that the inflammation–coagulation axis is also an important component of COVID-19-associated ALI/ARDS, the signaling pathways engaged by TNFα and thrombin to activate NF-κB may also be operative in ECs infected with SARS-CoV-2 [42,53,54,55,56,57,58]. Of note, although the scope of this review is restricted to the discussion of endothelial NF-κB activation due to its importance in mediating immune cell infiltration, similar mechanisms may exist in other cell types as well, contributing to the progression of ALI and other inflammatory diseases.

### 2.1. PKC Signaling to IKK/NF-κB

Protein kinase C (PKC) proteins are a highly conserved family of serine threonine kinases controlling broad cellular functions including gene expression, cell proliferation, and inflammation. PKC isoforms are grouped into three distinct subfamilies depending on their structure and activation mechanisms. Classical PKC isoforms (PKCα, PKCβI, PKCβII, and PKCγ) are activated by the lipid second messenger diacylglycerol (DAG) and calcium (Ca^2+^), whereas the novel PKC subfamily (PKCδ, PKCε, PKCθ, and PKCη) is activated by DAG, but not Ca^2+^. The atypical PKC subfamily (PKCζ and PKCι/λ) does not require DAG or Ca^2+^, and is instead activated by other lipid components [59,60]. Several PKC isoforms have been identified upstream of NF-κB signaling in the endothelium.

**Figure 7:**
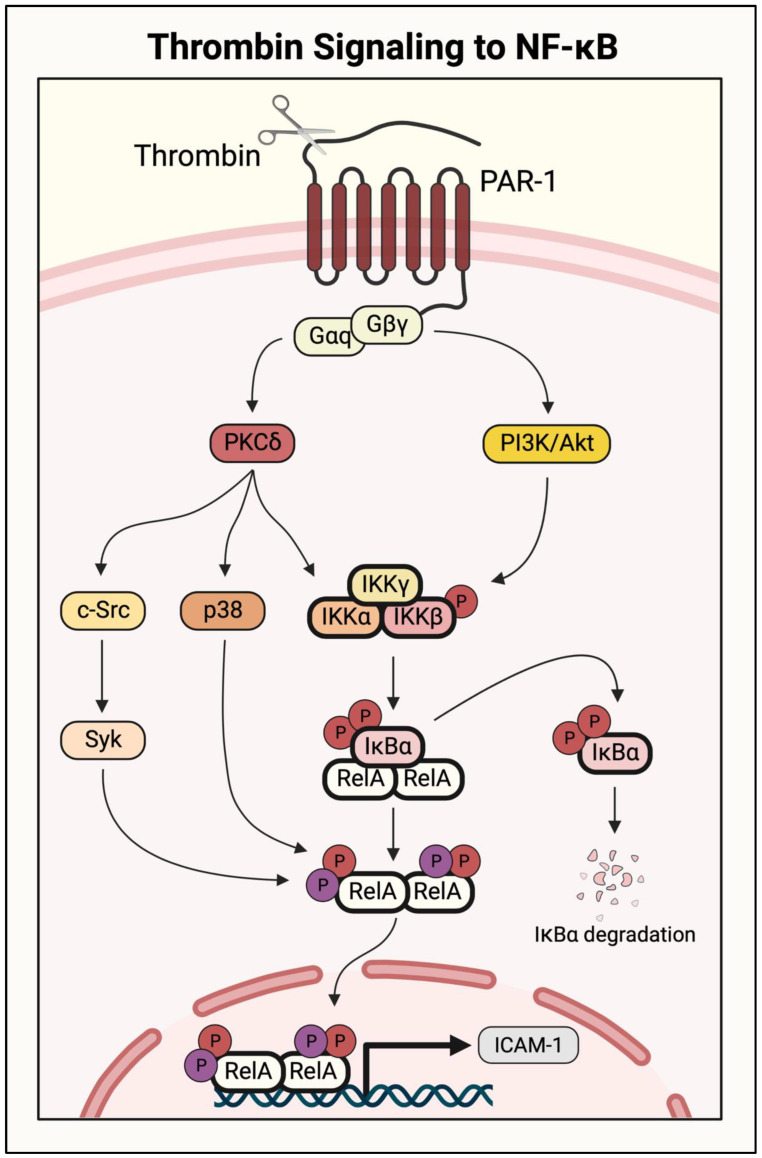
Thrombin-activated NF-κB pathway in endothelial cells. Thrombin activates its receptor, the G-protein-coupled receptor (GPCR) protease-activated receptor (PAR-1), by cleaving the extracellular domain to stimulate signaling. The Gβγ subunit activates phosphatidylinositol-3 kinase (PI3K)/Akt, which phosphorylates the IKK complex to activate canonical NF-κB signaling and induce RelA/p65 homodimer translocation to the nucleus. Gαq phosphorylates protein kinase C delta (PKCδ), which activates NF-κB by dual mechanisms. It induces IKK complex phosphorylation to release RelA/p65 for its translocation and DNA binding in the nucleus. It also engages p38 and c-Src/spleen tyrosine kinase (Syk) to phosphorylate RelA/p65 and enhance its transcriptional activity. Whereas p38 causes serine phosphorylation (red), c-Src and Syk cause tyrosine phosphorylation (purple).

**Figure 8:**
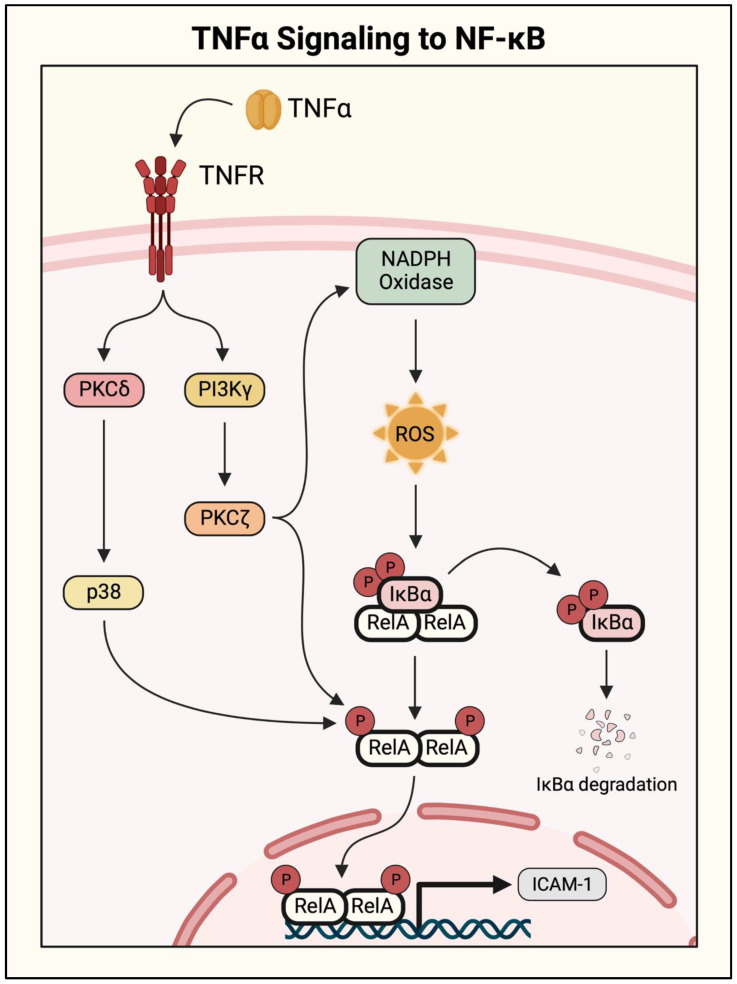
NF-κB activation by TNFα in endothelial cells. TNFα binding to its receptor, TNF receptor (TNFR), activates NF-κB by parallel pathways. It signals through PI3Kγ to activate PKCζ, which triggers NADPH oxidase to produce ROS. ROS then causes phosphorylation and degradation of IκBα and liberates RelA/p65 for its nuclear translocation and DNA binding. TNFα also initiates RelA/p65 serine phosphorylation through PI3Kγ/PKCζ and PKCδ/p38 pathways, amplifying its transcriptional activity.

Initial studies identifying a role for PKC isoforms examined the NF-κB pathway activity in response to PKC activators (phorbol esters and phorbol dibutyrate), and pharmacological and genetic approaches were used to identify the specific isoforms involved [19]. The procoagulatory protein thrombin activates NF-κB signaling by cleaving its receptor, protease-activated receptor 1 (PAR-1), on ECs. PAR-1 is a G-protein-coupled receptor (GPCR), and its activation signals through the G protein Gαq and Gβγ dimer. Activated Gαq couples to NF-κB activation via PKCδ (Figure 7). This novel PKC isoform interacts with the NF-κB pathway at two levels to induce the transcription of proinflammatory genes including *ICAM1*. It activates the pathway upstream by inducing IKKβ to phosphorylate IκBα, leading to its degradation and release of RelA/p65. It also acts downstream of IκBα to increase gene transcription by inducing RelA/p65 phosphorylation [61]. PKCδ has been implicated in TNFα-induced NF-κB activation as well. However, unlike with thrombin, the action of PKCδ in the TNFα response is restricted to RelA/p65 phosphorylation (Figure 8) [62].

Another PKC isoform activated by thrombin is the classical isoform PKCα. PKCα is activated by a thrombin-induced increase in intracellular calcium ([Ca^2+^]_i_) in ECs, leading to NF-κB activation. This process likely also engages PKCδ, but requires the participation of the Ca^2+^/calmodulin-dependent protein kinase kinase beta (CaMKKβ) and AMP-activated protein kinase (AMPK) as intermediate kinases [63,64]. The atypical PKC isoform PKCζ acts downstream of TNFα to activate NF-κB and promote ICAM-1 protein expression (Figure 8). In this case, PKCζ promotes the phosphorylation of IκBα as well as RelA/p65, facilitating NF-κB nuclear translocation and enhancing its transcriptional activity [65,66]. Furthermore, an in vitro model of vascular diabetic injury revealed a role for PKCβ in NF-κB signaling. Treatment with the PKCβ inhibitor attenuated IκBα degradation in response to high glucose; however, this has not yet been shown in the setting of thrombin- or TNFα-induced signaling [67]. These findings show how different stimuli can engage overlapping or divergent pathways to activate NF-κB. Broad-spectrum PKC inhibitors have elicited effective injury attenuation in mouse models of ALI; however, their clinical use may be complicated by vast off-target effects [59] as well as stimulus- and cell-specific roles of PKC isoforms in NF-κB regulation.

### 2.2. PI3K/Akt Signaling to IKK/NF-κB

Phosphatidylinositol-3 kinase (PI3K) enzymes are a family of protein kinases implicated in many disease states due to their critical roles in cell survival, growth, metabolism, and angiogenesis. PI3K proteins are activated by several upstream pathways including receptor-tyrosine kinases, small GTPases, or heterotrimeric G proteins, though specific PI3K isoforms contain interacting domains which allow them to transmit signals from distinct upstream mediators. For example, the class IA subgroup of catalytic isoforms (p110α, β, and δ) interact with regulatory subunits to bind growth factor receptors, whereas the class IB catalytic isoform p110γ and its associated regulatory subunits bind heterotrimeric G protein Gβγ subunits [68,69,70]. These distinctions in coupling allow the PI3K family proteins to mediate numerous cellular behaviors depending on stimulus and cellular context. In ECs, the p110α subunit of PI3K controls cell migration and angiogenesis during embryonic development, whereas the p110γ subunit and its downstream effector Akt have been implicated in the progression of ALI [71,72,73].

PI3K p110γ and Akt act downstream of both thrombin and TNFα to mediate NF-κB activation in ECs. Thrombin cleavage of PAR-1 causes activation of the IKK complex through parallel mechanisms: (1) via Gαq/PKCδ as described above, and (2) through Gβγ- dependent activation of PI3K p110γ (Figure 7). Activated p110γ engages its downstream effector Akt to stimulate IKKβ and induce proinflammatory gene transcription. In response to TNFα stimulation, p110γ acts upstream of PKCζ to activate NF-κB signaling, as shown by the impaired activation of PKCζ and NF-κB in p110γ knockout lung ECs (Figure 8) [73]. However, the cellular function of PI3K signaling in an inflammatory setting is not straightforward. A study of mouse microvascular ECs showed that pharmacological inhibition of PI3K/Akt signaling significantly increased TNFα-induced cytotoxicity, whereas NF-κB inhibition was protective [74]. Additionally, although PI3K/Akt pathway inhibition has been protective in multiple mouse models of ALI, PI3K/Akt signaling has also been shown to facilitate injury resolution [75,76,77]. In a septic mouse model, pharmacological inhibition or transgenic knockout of p110γ resulted in prolonged inflammation and vascular permeability [78]. Although not fully understood, these discrepancies may be due to additional functions of PI3K/Akt signaling apart from the regulation of NF-κB, as it has been shown to modulate metabolism, growth, proliferation, and survival [79]. Therapeutic modulation of PI3K/Akt signaling in ALI will require additional investigation to understand ideal intervention points in disease progression, preserving its cell viability and injury resolution phenotypes.

### 2.3. MAPK/p38 Signaling to IKK/NF-κB

The mitogen-activated protein kinase (MAPK) p38 has been shown to play an important role in thrombin- and TNFα-stimulated NF-κB signaling. Where thrombin signals through PKCδ and PI3K/Akt to activate the IKK complex, it also activates p38/MAPK via the PAR-1/Gαq/PKCδ axis to phosphorylate RelA/p65 at Ser^536^, thereby increasing its transcriptional activity (Figure 7) [19,61]. Although TNFα signals through a different receptor, it has been shown to activate a similar mechanism resulting in PKCδ/p38 activation and NF-κB dimer phosphorylation (Figure 7) [62]. NF-κB activity induced by both thrombin and TNFα requires p38/MAPK, as the expression of a dominant-negative p38 mutant prevented the induction of VCAM-1, ICAM-1, MCP-1, and IL-8 [19,21,61]. However, some groups have presented data that conflict with this finding, particularly in the case of TNFα-induced NF-κB signaling. Rajan et al. and Kuldo et al. both showed that p38/MAPK was not required for adhesion molecule expression in TNFα-stimulated ECs [80,81]. Although the role of p38/MAPK in EC inflammation has been debated, its global pharmacological inhibition has proven protective against ALI in a rat model of bacterial sepsis. Pretreatment with a pharmacological p38/MAPK inhibitor reduced histological lung injury, as well as the serum levels of proinflammatory cytokines IL-6 and TNFα [82]. Therefore, p38/MAPK inhibition may prove an effective therapeutic intervention in ALI progression, but cell-specific contributions remain to be fully understood.

### 2.4. Calcium Signaling to IKK/NF-κB

Calcium (Ca^2+^) is an important second messenger in mammalian cells with diverse roles in cell fate decisions, metabolism, and gene regulation [83]. In the activated endothelium, increases in [Ca^2+^]_i_ stimulate various downstream signaling pathways, inducing NF-κB nuclear translocation and activation. Intensive studies utilizing genetic and pharmacological interventions identified the pathways linking an increase in [Ca^2+^]_i_ to NF-κB activation. The increase in [Ca^2+^]_i_ by proinflammatory mediators occurs in two phases. In the first transient peak, Ca^2+^ is released from stores in the endoplasmic reticulum (ER). For example, thrombin causes the ER-store Ca^2+^ release through sequential events, including the activation of phospholipase C β (PLCβ), the generation of inositol-1,4,5-triphosphate (IP_3_) and diacylglycerol (DAG), and the interaction of IP_3_ with its receptor (IP_3_R). IP_3_R is the main Ca^2+^-release channel on the ER membrane, and its activation shuttles Ca^2+^ out of the ER. This transient peak in [Ca^2+^]_i_ is followed by a second, more sustained peak as Ca^2+^ enters the cell through a process called store-operated Ca^2+^ entry (SOCE). Upon depletion of ER Ca^2+^ stores, the Ca^2+^-sensing protein stromal interacting molecule-1 (STIM1) initiates SOCE by clustering on the ER membrane and interacting with transient receptor potential canonical (TRPC) channels on the plasma membrane. Binding and activation of TRPC channels, primarily TRPC1 and TRPC4 in ECs, facilitates Ca^2+^ entry into the cell and further increases [Ca^2+^]_i_. Experiments utilizing TRCP1 overexpression or *TRPC4*^−/−^ EC revealed their requirement for thrombin-induced Ca^2+^ influx and subsequent increases in [Ca^2+^]_i_ [64,84]. In addition to TRPC1 and TRPC4, ECs express TRPC6, which also regulates Ca^2+^ entry [85,86,87]. However, unlike TRPC1 and TRPC4, TRPC6 mediates receptor-operated Ca^2+^ entry (ROCE), which is activated by DAG and does not require store depletion [87]. Given that TRPC6-driven ROCE can promote TRPC1-mediated SOCE [88], the cooperation between these TRPCs may represent a mechanism to augment and prolong the increase in [Ca^2+^]_i_, and the downstream signaling in EC.

The increase in [Ca^2+^]_i_ has been shown to cause NF-κB activation by multiple mechanisms. In one pathway, the thrombin-induced increase in Ca^2+^ stimulates RelA/p65 nuclear translocation and transactivation by a CAMKKβ/AMPK/PKCδ/p38-dependent signaling cascade. Activation of this pathway serves dual functions; p38/MAPK induces NF-κB activation and proinflammatory signaling, and in parallel, initiates a self-limiting mechanism by inactivating STIM1 to terminate Ca^2+^ entry [64,89]. These findings suggest that targeting this pathway to limit NF-κB in the setting of inflammatory disease might produce unfavorable results by eliminating an important negative regulatory function. In contrast, the Ca^2+^-sensitive kinase PKCα occupies a central position in a feed-forward mechanism linking increased [Ca^2+^]_i_ and NF-κB activation. In this model, thrombin increases [Ca^2+^]_i_ levels_,_ activating PKCα to induce NF-κB signaling. NF-κB-mediated gene transcription causes expression of new TRPC1 protein, facilitating more Ca^2+^ entry and potentiating NF-κB activation. Importantly, Ca^2+^ chelation or the inhibition of PKCα was sufficient to attenuate this amplifying cascade in cultured ECs, raising their potential as promising therapeutic targets to limit proinflammatory signaling [63].

Although, the rise in [Ca^2+^]_i_ is rapid and crucial for NF-κB activation in response to thrombin, NF-κB activation by TNFα occurs in a Ca^2+^-independent manner [90,91]. However, it should be noted that activation of the coagulation cascade usually co-occurs with TNFα generation in the setting of inflammatory disease. The crosstalk between procoagulant and proinflammatory signaling is particularly important in Ca^2+^-mediated signaling, where thrombin and TNFα are reported to have synergistic effects on Ca^2+^ entry. TNFα alone causes a very slow and weak increase in [Ca^2+^]_i_, but pretreatment of ECs with TNFα before thrombin stimulation not only augments, but also extends the Ca^2+^ peak from 3 min to 10 min [91]. A further study revealed that TNFα induces the NF-κB-mediated expression of TRPC1, thus facilitating the indirect and delayed Ca^2+^ entry [90]. These findings not only implicate Ca^2+^ signaling in a vicious cycle of intensifying inflammation, but also signify the cooperation of proinflammatory and procoagulant signaling. Thus, interrupting this feed-forward mechanism to limit uncontrolled inflammation may have therapeutic potential against ALI.

### 2.5. Actin–Myosin Interaction and Small GTPase Signaling to IKK/NF-κB

**Figure 9:**
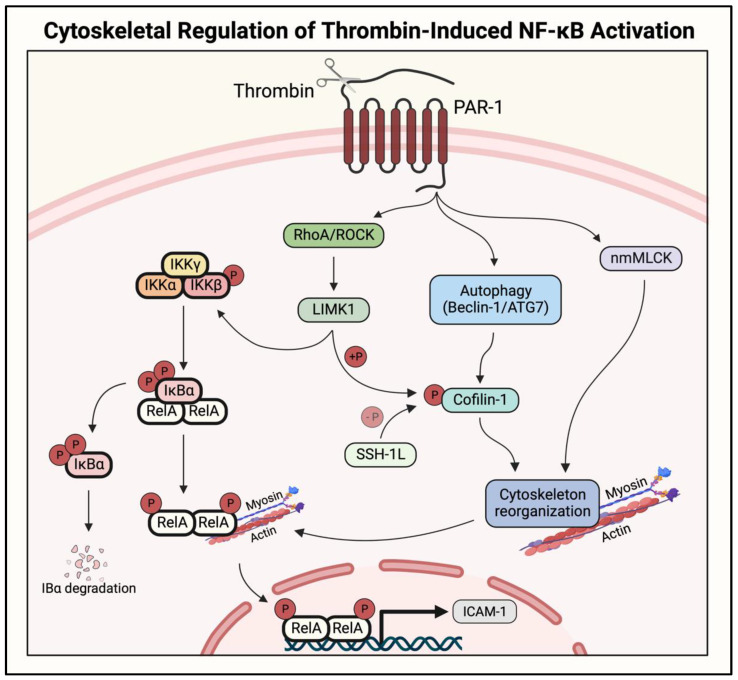
Small GTPase/cytoskeletal regulation of thrombin-induced NF-κB activation. Thrombin activation of PAR-1 stimulates RhoA/Rho-associated kinase (ROCK) signaling, nonmuscle myosin light-chain kinase (nmMLCK) activity, and autophagic flux. RhoA/ROCK activates LIM kinase 1 (LIMK1), which acts to phosphorylate IKKβ, causing release of RelA/p65 from IκBα. In parallel, LIMK1 causes phosphorylation and inactivation of cofilin-1 to induce actin cytoskeleton reorganization. Thrombin also activates nmMLCK, which acts in concert with LIMK1/cofilin-1 to promote actin–myosin interaction. Generation of actin–myosin stress fibers by this mechanism facilitates the nuclear translocation of released RelA/p65. Induction of autophagy via Beclin-1 and ATG7 also contributes to cofilin-1 phosphorylation and stress fiber formation. Cofilin-1 phosphorylation is tightly regulated by engagement of slingshot-1Long (SSH-1L), a cofilin-1 phosphatase.

The regulation of small GTPases and actin cytoskeletal dynamics represents additional stimulus-specific mediators of NF-κB signaling in ECs. The RhoA/Rho-associated kinase (ROCK) pathway is required for thrombin-induced NF-κB activation and ICAM-1 expression, and inhibition of Rho or ROCK prevented these responses induced by thrombin, but not by TNFα [92,93]. The RhoA/ROCK pathway was found to activate NF-κB signaling through IKK complex activation, resulting in IκBα degradation and RelA/p65 nuclear translocation (Figure 9) [92]. TNFα-stimulated NF-κB signaling does not involve RhoA activation; instead, it is mediated by a different small GTPase Rac1. Expression of a dominant-negative Rac1 mutant (N17Rac1) inhibited the TNFα-induced expression of adhesion molecules ICAM-1 and VCAM-1 via inhibition of the NF-κB pathway. Interestingly, N17Rac1 did not prevent the DNA binding activity of NF-κB, and instead, inhibited its transactivation potential [94]. Additional studies from the same group showed that Rac1 also regulated the TNFα-induced secretion of MCP-1 via NF-κB [95]. Rho GTPases mediate actin cytoskeleton dynamics and cell adhesion, and RhoA and Rac1 are known to play antagonistic roles in this regulation [96]. The stimulus-specific engagement of RhoA and Rac1 to activate NF-κB may be related to the differential effects of thrombin and TNFα on the actin cytoskeleton.

The actin–myosin interaction (actin stress fiber formation) occurs rapidly after thrombin stimulation, leading to EC contraction and destabilization of cell–cell junctions; however, this is a much slower process in TNFα-stimulated ECs [97]. Actin stress fiber formation downstream of RhoA/ROCK signaling was found to be essential for the thrombin-induced nuclear translocation of RelA/p65 after IκBα degradation. Consistent with the role of RhoA, the pharmacological modulation of the actin cytoskeleton to either stabilize or destabilize the actin stress fibers each prevented thrombin-induced, but not TNFα-induced RelA/p65 nuclear translocation [97]. Later, cofilin-1, an actin-binding protein that promotes actin depolymerization, was identified as a critical regulator of the dynamic changes in the actin cytoskeleton downstream of thrombin-induced RhoA/ROCK activation [98]. Consistent with this, depletion of cofilin-1 or modulation of its activity to stabilize or destabilize the actin cytoskeleton each attenuated thrombin-induced but not TNFα-induced NF-κB activity and ICAM-1 expression by impairing RelA/p65 nuclear translocation (Figure 9) [98]. These studies revealed that cofilin-1 occupies a central position in the RhoA-actin pathway, mediating thrombin-induced RelA/p65 nuclear translocation in ECs [98]. Further studies identified that LIM kinase 1 (LIMK1), a cofilin kinase, and slingshot-1Long (SSH-1L), a cofilin phosphatase, are engaged by thrombin, but not TNFα, to regulate the dynamic changes in the cofilin-1 phosphorylation/inactivation status. LIMK1 acts downstream of RhoA/ROCK to induce phosphorylation, thereby inactivating Cofilin-1 and resulting in stress fiber formation and RelA/p65 nuclear translocation (Figure 9). Importantly, LIMK1 also acts in parallel to phosphorylate IKKβ and cause the release of RelA/p65 from IκBα [99]. Unlike LIMK1, the action of SSH-1L occurs downstream of cofilin-1 phosphorylation and is restricted to RelA/p65 nuclear translocation [99]. Together, these studies unequivocally establish that the RhoA/ROCK/LIMK1 pathway is selectively engaged by thrombin to promote NF-κB signaling, both by activating the IKKβ to release RelA/p65 and by inducing stress fiber formation to facilitate nuclear localization of the released RelA/p65 (Figure 9). These findings have implications for the specific targeting of the RhoA/ROCK/LIMK1 pathway as a useful strategy for controlling inflammatory responses associated with intravascular coagulation.

The formation of actin stress fibers involves the crosslinking of actin filaments and myosin bundles, and a crucial role for the actin-binding protein, nonmuscle myosin light-chain kinase (nmMLCK), was identified in thrombin-induced NF-κB activation [100,101]. Mice deficient in nmMLCK were significantly protected from lung ICAM-1 expression and PMN recruitment upon an intraperitoneal thrombin challenge, and nmMLCK inhibition or knockdown in cultured ECs attenuated NF-κB activation and the expression of ICAM-1 and MCP-1 [100]. Consistent with the role of the actin cytoskeleton in RelA/p65 translocation, nmMLCK depletion prevented RelA/p65 nuclear localization without affecting IκBα degradation (Figure 9) [98,100]. These data corroborate the role of actin–myosin contractile forces in mediating thrombin-induced NF-κB activation. However, they exclude the role of the actin–myosin interaction in TNFα-induced RelA/p65 translocation, and raise the question of whether its nuclear localization caused by TNFα occurs passively or is facilitated by other mechanisms.

The differential regulation of Rho GTPases in NF-κB activation highlight the importance of understanding the up- and downstream modulators of the NF-κB pathway; both thrombin and TNFα can activate RhoA (though with different kinetics), but only thrombin engages RhoA/ROCK signaling to activate NF-κB-mediated gene expression [93]. The signaling role of [Ca^2+^]_i_ may contribute to this stimulus-specific modulation of NF-κB by the RhoA-actin pathway. This possibility is supported by the findings that thrombin rapidly induces RhoA-mediated polymerization of actin filaments, promoting the IP_3_R–TRPC1 interaction to facilitate Ca^2+^ entry [86]. Furthermore, increased [Ca^2+^]_i_ causes activation of both nmMLCK and RhoA, leading to stress fiber formation and increased endothelial permeability [84]. However, it remains to be determined whether Ca^2+^ signaling and the RhoA-actin pathway collaborate in the activation of NF-κB, or if they act through independent mechanisms.

The critical role of RhoA/ROCK signaling in thrombin-induced NF-κB activation and EC inflammation suggests that ROCK inhibition might be beneficial in clinical ALI treatment. The clinically approved ROCK inhibitor fasudil, or Y-27632, has been shown to be well-tolerated in human patients and attenuates inflammation associated with another inflammatory lung condition, pulmonary arterial hypertension [102]. Fasudil may prove beneficial in ALI/ARDS patients as well; however, further studies are required to understand the interplay between ALI heterogeneity and the stimulus-specific role of ROCK to maximize its effectiveness.

### 2.6. Oxidant Signaling to IKK/NF-κB

Reactive oxygen species (ROS) are generated as a byproduct of normal cell function and play important roles in homeostasis and cell signaling, including NF-κB activation. Oxidants have been found to mediate NF-κB signaling in response to several different stimuli, and their function in response to TNFα is the most well-characterized. In ECs, TNFα signals through PI3Kγ to activate PKCζ, which then activates NF-κB signaling in two parallel mechanisms. It directly induces RelA phosphorylation, and it also causes the activation of NADPH oxidase, triggering ROS generation and subsequent IκBα degradation (Figure 8). The requirement for NADPH oxidase was shown through experiments with mice deficient in NADPH oxidase subunits p47^phox^ or gp91^phox^. Both lungs and lung microvascular ECs (LMVECs) isolated from p47^phox^ and gp91^phox^ knockout mice showed attenuated NF-κB activation and ICAM-1 expression [73,103]. Furthermore, a recent study showed the effectiveness of the antioxidant resveratrol in attenuating TNFα-induced ROS and p38 MAPK/NF-κB signaling [104]. Interestingly, thrombin-stimulated NF-κB signaling is mediated by oxidant generation, but is independent of NADPH oxidase [19]. Thrombin, instead, stimulates mitochondria to produce mitochondrial ROS via IP_3_R/Ca^2+^ signaling. Generated mitochondrial ROS triggers NF-κB activation, causing ICAM-1 expression and leukocyte adhesion to the vascular endothelium. This effect was corroborated by inhibition of NF-κB in the presence of a ROS scavenger, and intact NF-κB signaling in cells lacking NADPH oxidase subunit gp91^phox^ showed that this regulation was specific to mitochondrial ROS [105]. Therefore, both thrombin and TNFα stimulation of the NF-κB pathway are mediated by ROS generation; however, the source of ROS and mechanism of action appear to be stimulus-specific. Studies of ALI/ARDS patients have observed increased oxidant levels and decreased antioxidants in injured lungs, with higher nitric oxide levels correlating with mortality [106,107,108]. However, antioxidant therapy has led to mixed results in patient trials [109], potentially due to the possible toxicity of antioxidants owing to their inherent ability to produce ROS, especially in the highly oxidizing environment of injured lung.

### 2.7. Tyrosine Kinase Signaling to IKK/NF-κB

The importance of serine phosphorylation in NF-κB signaling has been extensively studied as the activity of IKK complex proteins, IκBα, and NF-κB subunits are modulated by serine kinases. Later studies reported that tyrosine kinases, another important family of signaling proteins, also play a vital role in NF-κB pathway regulation. Early studies investigating the role of tyrosine kinases employed broad pharmacological inhibitors and showed their involvement in the TNFα-induced NF-κB activation and expression of target genes *VCAM1*, *ICAM1*, and *SELE* (E-selectin) [110]. The action of tyrosine kinases in promoting NF-κB activation was found to occur at the level of NF-κB DNA binding [110]. Studies using robust genetic approaches to disable specific tyrosine kinases identified c-Src and spleen tyrosine kinase (Syk) as key regulators of NF-κB activity [111,112]. Analysis of the NF-κB activation pathway showed that depleting/inhibiting c-Src or Syk attenuated NF-κB transcriptional activity and expression of its target genes, but had no effect on IκBα degradation, RelA/p65 nuclear translocation, and DNA binding caused by thrombin (Figure 7) [111,112]. Loss of the c-Src or Syk activity/level also failed to inhibit RelA/p65 phosphorylation at Ser^536^, which is implicated in conferring transcriptional competency to RelA/p65 DNA-bound NF-κB [111,112]. The action of these kinases in regulating NF-κB activity is not stimulus-specific, as they also mediate the NF-κB activity in response to a variety of agonists including TNFα, LPS, and PMA (K.M. Bijli and S.A. Slavin, unpublished results). Further analysis showed that both c-Src and Syk control NF-κB transcriptional activity by a novel mechanism that relies on tyrosine phosphorylation of RelA/p65 (Figure 7) [111,112]. Consistent with this, both c-Src and Syk associate with and phosphorylate RelA/p65 in ECs stimulated with thrombin [111,112].

There is some evidence that Syk is engaged downstream of c-Src by thrombin [112]; however, it is unclear whether c-Src and Syk associate with RelA/p65 in a sequential manner such that the binding of one is required for the other, or if they can bind to RelA/p65 independently of each other. It also remains to be determined whether c-Src and Syk phosphorylate the same or different tyrosine residue(s). Additional studies are needed to address these questions. Interestingly, the c-Src/Syk pathway is linked to PKC and signals downstream of PKCδ to activate NF-κB (Figure 7) [112]. The coexpression of the constitutively active PKCδ mutant and a dominant-negative Syk mutant showed that this tyrosine phosphorylation is required for the proinflammatory effect of PKCδ [111,112]. It is unclear how tyrosine phosphorylation of RelA/p65 regulates its transcriptional activity, and identifying the phosphorylation site may reveal key mechanistic insights. Additionally, PKCδ induces serine phosphorylation of RelA/p65 through p38/MAPK, but whether this occurs in parallel with c-Src/Syk or in the same pathway is unknown. Depletion of c-Src and Syk did not affect RelA/p65 Ser^536^ phosphorylation, and thus, they do not act upstream of p38/MAPK, but the converse cannot be excluded [112]. It is likely these pathways act in parallel because they induce different types of RelA/p65 phosphorylation (i.e., serine vs. tyrosine); however, this remains to be investigated.

Another tyrosine kinase that is required for NF-κB activation is proline-rich tyrosine kinase 2 (Pyk2) [113]. Pyk2 is a calcium-dependent protein tyrosine kinase and belongs to the focal adhesion kinase family [113]. It is an important component of EC inflammation caused by thrombin and TNFα, and mediates this response by activating NF-κB. Unlike c-Src and Syk, Pyk2 activates IKK to induce the release, nuclear translocation, and DNA binding and phosphorylation of RelA/p65. Additionally, the Pyk2/IKK axis increases the transactivation potential of RelA/p65 by inducing its phosphorylation at Ser^536^ [113]. However, it is unclear if Pyk2, like c-Src and Syk, also contributes to the transcriptional activity by increasing tyrosine phosphorylation of RelA/p65. Given that c-Src can function downstream of Pyk2 [114], such a possibility cannot be excluded. The involvement of both Pyk2/IKK and p38 MAPK in thrombin-induced Ser^536^ phosphorylation of RelA/p65 suggests that these pathways may be linked in mediating this event. However, the finding that the thrombin activation of p38 MAPK is not affected in Pyk2- and IKK-depleted cells argues against the involvement of Pyk2/IKK/p38 MAPK in RelA/p65 Ser^536^ phosphorylation. Moreover, the inability of p38 MAPK, unlike Pyk2/IKK, to mediate IκBα degradation and RelA/p65 nuclear translocation further suggests that these pathways act independently to mediate RelA/p65 phosphorylation [113]. Paradoxically, the Pyk2/p38 MAPK axis is implicated in LPS-induced NF-κB [115], indicating a stimulus-specific engagement of p38 MAPK by Pyk2 in ECs, and showing a fundamental signaling difference between thrombin and other receptors (GPCR versus TLR4). These reports also highlight the diverse mechanisms engaged by tyrosine kinases to activate NF-κB in ECs.

### 2.8. ER and Mitochondrial Chaperone Signaling to IKK/NF-κB

Communication between the endoplasmic reticulum (ER) and mitochondrion, two multifunctional cellular organelles, is central to many cellular processes (Ca^2+^ homeostasis, energy metabolism, cell survival, and lipid metabolism) [116,117]. This is achieved through a specialized region of close contact (~10–25 nm) between the ER and mitochondria, termed the mitochondria-associated ER membrane (MAM) [116,118]. MAMs harbor many chaperones and several key calcium channels involved in intracellular Ca^2+^ homeostasis and phospholipid exchange [117,118,119]. BiP/GRP78 (binding immunoglobulin protein/78-kDa glucose-regulated protein) and Mortalin/GRP75 are important components of MAMs [120]. BiP/GRP78 is an ER chaperone that is upregulated during the unfolded protein response (UPR) to facilitate protein refolding, assembly, and translocation of newly synthesized polypeptides across the ER membrane, and the targeting of misfolded proteins for ER-associated degradation (ERAD) [121]. Mortalin/GRP75 is a mitochondrial chaperone that plays essential roles in protein folding, mitochondrial import, intracellular trafficking, and proliferation [122,123].

Aberrant regulation of BiP/GRP78 and Mortalin/GRP75 is associated with many chronic inflammatory diseases including type 2 diabetes, atherosclerosis, cancer, and several neurodegenerative diseases [124,125,126,127,128,129]. More recently, these chaperones have been implicated in ALI pathogenesis [130,131]. The level of BiP/GRP78 is increased in the injured lung, and targeting it by subtilase cytotoxin (SubAB), a serine protease produced by Shiga-toxigenic *E. coli* that specifically cleaves and inactivates BiP/GRP78 [132], reduced lung inflammation and improved lung compliance in mice after LPS inhalation, whereas the inactive mutant of BiP/GRP78 (SubA_A272_B) had no effect [130]. Similarly, overexpression of a dominant-negative mutant of BiP/GRP78 in the lung endothelium attenuated LPS-induced lung inflammatory responses [130], indicating a pathogenic role of endothelial BiP/GRP78 in ALI. Consistent with this, in vitro studies showed that BiP/GRP78 is necessary for NF-κB signaling and EC inflammation induced by thrombin, but not TNFα [130,133]. Knockdown of BiP/GRP78 blocked thrombin-induced IKKβ phosphorylation, preventing IκBα phosphorylation/degradation, RelA/p65 serine phosphorylation, and its nuclear translocation. Depletion of BiP/GRP78 also limited Ca^2+^ release from ER stores in response to thrombin, a key event mediating NF-κB activation in ECs. Interestingly, inactivation of BiP/GRP78 by SubAB inhibited NF-κB signaling by a different mechanism. The Sub-AB treatment did not affect IκBα degradation or the nuclear localization of RelA/p65, and instead prevented its DNA binding [133]. These data suggest that intact BiP/GRP78 might facilitate NF-κB activation at multiple steps along the pathway, possibly through the cooperation of distinct protein subdomains. Removing the BiP/GRP78 protein inhibits the entire NF-κB pathway, whereas the BiP cleavage product(s) formed by the SubAB treatment appear to maintain some of their function, but are unable to entirely recapitulate the activity of the full-length protein. This is consistent with the finding that BiP/GRP78 is constitutively associated with RelA/p65 (F. Fazal, unpublished results) and points to the possibility that this association is lost or altered when BiP/GRP78 is cleaved by SubAB. It is likely that full-length BiP/GRP78 can also bind IκBα while it is associated with RelA/p65, and this association may render IκBα accessible to IKKβ for its phosphorylation/degradation and facilitate RelA/p65 binding to the DNA in the nucleus. Further studies focused on identifying the interacting domains of BiP/GRP78 with RelA/p65 and IκBα are required to determine the mechanistic basis of the BiP/GRP78 regulation of RelA/p65 release in the cytoplasm and DNA binding in the nucleus.

Like BiP/GRP78, Mortalin/GRP75 also plays a causal role in ALI [131]. Mice treated with the Mortalin/GRP75 inhibitor MKT-077 (a cationic rhodacyanine dye) showed attenuated lung vascular inflammation and leakage and improved lung function after LPS inhalation. Importantly, MKT-077, when administered therapeutically, was equally effective in protecting against LPS-induced ALI. The LPS-induced loss of VE-Cadherin was also prevented in these mice [131], indicating the importance of endothelial Mortalin/GRP75 in lung vascular injury. Interestingly, Mortalin/GRP75 shares several similarities with BiP/GRP78 in regulating NF-κB activation in ECs. Mortalin/GRP75 constitutively associates with RelA/p65, and depleting or inhibiting it prevented the thrombin-induced increase in [Ca^2+^]_i_ and RelA/p65 DNA binding [131]. However, unlike BiP/GRP78 knockdown, depletion of Mortalin/GRP75 does not affect IκBα degradation, nor the nuclear translocation or phosphorylation of RelA/65 [131]. It is unclear whether BiP/GRP78 and Mortalin/GRP75 bind RelA/p65 together or separately; however, their similar roles in RelA/p65 DNA binding suggest the potential for a functional BiP–Mortalin–RelA/p65 complex. It is likely that upon stimulation with thrombin, the BiP/GRP78 and/or Mortalin/GRP75 activity in this complex might recruit additional regulatory proteins to facilitate RelA/p65 DNA binding. Defining BiP/GRP78- and Mortalin/GRP75-binding domains in RelA/p65 and determining the localization of the BiP–Mortalin–RelA/p65 complex in resting and stimulated ECs will be key to address these possibilities. The shared roles of BiP/GRP78 and Mortalin/GRP75 in mediating the [Ca^2+^]_i_ increase and EC inflammation support the potential for a broader role of MAMs in NF-κB regulation. Further investigation into the role of the MAM proteins will be critical to understand the interplay between MAM integrity and the NF-κB signaling.

### 2.9. Autophagy Protein Signaling to IKK/NF-κB

Autophagy is a catabolic process that recycles intracellular components to be used for energy production, and its dysregulation has been implicated in numerous diseases [134,135]. Studies have shown that autophagy is induced in thrombin-treated EC and mediates NF-κB activation, and pharmacological and genetic approaches revealed a role for autophagy in thrombin-induced stress fiber formation [136,137,138]. Inhibiting autophagy by the depletion of key autophagy proteins Beclin-1 or ATG7 attenuated cofilin-1 phosphorylation in thrombin-treated ECs, preventing stress fiber-mediated nuclear translocation of RelA/p65 (Figure 9). Accordingly, knockdown of these proteins prevented thrombin-induced NF-κB activity and proinflammatory gene expression in ECs, though it is unclear whether Beclin-1 or ATG7 is linked to cofilin-1 phosphorylation via activation of the RhoA/ROCK/LIMK1 pathway or inactivation of slingshot-1Long (SSH-1L), a cofilin phosphatase. Because RhoA/ROCK/LIMK1 also activates RelA/p65 release from IκBα by phosphorylating/activating IKKβ, it is likely that Beclin-1 and ATG7 regulate cofilin-1 phosphorylation via SSH-1L inactivation given that Beclin-1 or ATG7 depletion had no effect on IκBα degradation (Figure 9) [99,136,137]. Intriguingly, however, the Beclin-1 and ATG7 regulation of NF-κB differs in one aspect. Although the Beclin-1 knockdown inhibited RelA/p65 phosphorylation at Ser^536^, the ATG7 knockdown had no effect on this event. One potential explanation could be that Beclin-1, unlike ATG7, engages RhoA/ROCK/LIMK1, either solely or in combination with SSH-1L, to control the phosphorylation of both RelA/p65 and cofilin-1. However, this possibility remains to be clarified. Nevertheless, the shared mechanism of RelA/p65 nuclear translocation by Beclin-1 and ATG7 implicate autophagy as a key component of EC inflammation.

Despite the role of critical autophagy proteins Beclin-1 and ATG7 in mediating RelA/p65 translocation and EC inflammation, autophagy inhibition in mouse models of ALI has yielded mixed results. Our studies using an aerosolized bacterial LPS inhalation mouse model of ALI revealed enhanced autophagy in the lung as evidenced by increased expression of ATG5, and treatment with the autophagy inhibitor 3-methyladenine (3-MA), either prophylactically or therapeutically, was sufficient to attenuate lung PMN infiltration, lung vascular leakage, and tissue edema [139]. In accordance with these findings, other studies showed protective effects of autophagy inhibition with hydrogen sulfide or 4-phenyl butyric acid against ALI in mice caused by intratracheal administration of LPS [140,141]. Similarly, targeting autophagy also protected against ALI caused by avian influenza A H5N1 infection or mechanical ventilation [142,143]. However, there are several contrasting reports where autophagy has been shown to exert a protective role in various models of ALI [144,145,146,147,148]. The differential role of autophagy in ALI and other diseases may derive from accelerated or impaired autophagy, which causes imbalances in the expression or activation of autophagic proteins depending upon the model system or cell/tissue type [146,149,150].

### 2.10. MTOR Signaling to IKK/NF-κB

The mechanistic target of rapamycin (MTOR), a serine/threonine kinase belonging to the phosphatidylinositol kinase-like kinase family, is a major regulator of cell growth, protein synthesis, and metabolism [134]. MTOR functions in two distinct multiprotein complexes, MTOR complex 1 and 2 (MTORC1 and MTORC2), to exert these functions. The defining subunits of MTORC1 and MTORC2 are the rapamycin-sensitive regulatory-associated protein of MTOR (Raptor) and the rapamycin-insensitive companion of MTOR (Rictor), respectively. These complexes activate different downstream effectors and mediate distinct cellular functions [69,134,151,152,153,154]. Our studies have shown that MTOR limits NF-κB EC inflammation by dampening activation. MTOR was found to inhibit thrombin-induced IKKα/β phosphorylation, and thereby attenuates RelA/p65 nuclear translocation and proinflammatory gene expression [155]. Seemingly paradoxical findings showed that thrombin activates MTOR via PKCδ- and PI3K/Akt-dependent mechanisms, in contrast with their described functions as NF-κB pathway activators (Figure 7) [72,156]. Further investigation revealed MTOR as an endogenous self-limiting mechanism in the endothelium to ensure transient NF-κB activation. MTOR is activated by thrombin in a biphasic manner, with rapid activation and inactivation followed by a delayed, persistent re-activation [72]. Intriguingly, NF-κB activation peaks between these two phases when MTOR activity is at its lowest, and time-course analyses suggest that this reciprocal relationship ensures tight regulation to restrict the duration and intensity of NF-κB activation [72,155]. Pharmacological inhibition of MTOR led to precipitous, augmented, and prolonged activation of NF-κB after a thrombin challenge [72]. Importantly, MTOR appears to be a general regulator of NF-κB in the endothelium as it also suppresses NF-κB activity induced by TNFα, though it has not been confirmed whether TNFα also activates MTOR in a biphasic manner [155]. It should be emphasized that the above findings are based on studies using rapamycin or MTOR knockdown [72,155]. Although these studies may suggest a role of MTORC1, additional studies using Raptor or Rictor knockdown are required to unequivocally determine the contribution of MTORC1 vs. MTORC2 in NF-κB regulation. Another study identified a role for MTOR signaling in attenuating TNFα-induced NF-κB phosphorylation via a PKCδ/p38-dependent pathway [62]. This study showed that the knockdown of Raptor or Rictor each reduced NF-κB phosphorylation, implicating the role of both MTORC1 and MTORC2 in NF-κB activation. These data highlight the important role of MTOR in limiting NF-κB signaling in the endothelium, and support its potential as a therapeutic target to treat ALI and other inflammatory conditions.

MTOR plays major roles in the inflammatory signaling of other cell types; however, it appears to have either pro- or anti-inflammatory functions depending on the cellular context [157]. In monocytes/macrophages, MTOR limits inflammation by inhibiting NF-κB proinflammatory signaling and inducing STAT-3 anti-inflammatory gene expression, whereas it promotes NF-κB signaling in PMNs [158,159]. The reason for its cell-specific function is unclear, but the inherent regulatory mechanisms of MTOR signaling may contribute to this discrepancy. The divergent roles of MTOR in inflammatory regulation may be due, in part, to the relative expression and activation of MTOR complexes in different cell types. In a two-hit model of intratracheal or intravenous LPS exposure followed by mechanical ventilation, NF-κB signaling was upregulated and MTORC2 signaling and protein expression were reduced in whole lung lysates from injured rats and pigs [160]. Corroborating these findings, a study employing mice with the endothelial-restricted knockout of MTOR, Raptor (MTORC1), or Rictor (MTORC2) found that the knockout of MTOR complexes augmented lung injury caused by sepsis [62]. In contrast, a separate study identified increased MTORC1 signaling in the mouse lung epithelium after injury induced by microbial sepsis and mechanical ventilation [161]. These data suggest a cell- and complex-specific role for MTOR signaling in ALI. Targeting MTOR may be a potential therapeutic intervention to limit the detrimental effects of inflammation during ALI, but it will be important to gain a deeper understanding of the regulatory mechanisms and cell-type-specific effects of MTOR in the lung.

One important downstream function of MTOR activity that might contribute to its divergent functions is its negative regulation of autophagy. MTORC1 and MTORC2 have both been identified as autophagy inhibitors, though they act via distinct downstream mediators [162]. Further studies will be required to understand the contribution of autophagy inhibition to the protective effects of MTOR in the endothelium, especially considering the inconsistent role of autophagy in ALI animal models. Autophagy is only one of a number of functions downstream of MTOR, and hence, autophagy is unlikely to influence all aspects of NF-κB regulation by MTOR. Indeed, MTOR inhibits activation of IKK in response to thrombin, whereas Beclin-1 or ATG7 depletion did not affect IκBα degradation, and thus, autophagy is unlikely to act upstream of this step in the NF-κB pathway (Figure 9) [72,136,137]. Nevertheless, the inhibition of autophagy might enable MTOR to limit NF-κB signaling by impeding RelA/p65 nuclear localization. However, the precise interplay between MTOR and autophagy in NF-κB regulation and how it is influenced by the cellular context and disease models require further investigation.

## 3. Therapeutic Potential and Problems of Targeting NF-κB in ALI

**Figure 10:**
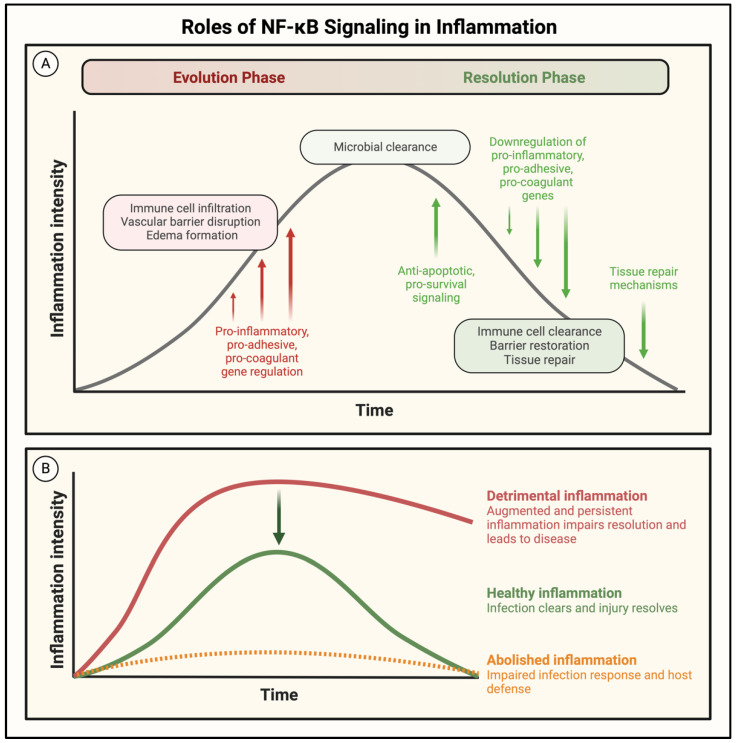
Roles of NF-κB signaling in evolution and resolution of inflammation. (**A**) In response to infection and injury, NF-κB activation initiates transcription of proinflammatory, proadhesive, and procoagulant genes. As resolution begins, changes/decline in NF-κB signaling promote tissue repair by inducing anti-inflammatory, antiadhesive, anticoagulant, and prosurvival mechanisms. (**B**) Dampening inflammation: an effective strategy to treat inflammatory conditions. Healthy inflammation (green) is self-limiting and resolves after infection is eliminated and tissue is repaired. However, in many disease conditions (red) inflammation becomes exuberant and persistent, and exerts detrimental effects on host tissues. Abolishing NF-κB/inflammation (orange) is counterproductive as it would impair the host defense response and tissue repair. Developing effective therapeutics to treat inflammatory conditions relies on selectively targeting detrimental inflammation while leaving healthy inflammation intact.

NF-κB activates an array of transcriptional programs broadly influencing cell behavior, and these heavily conserved roles establish it as a critical modulator of cell function. NF-κB signaling is tightly regulated, and in most cases, a proinflammatory stimulus activates NF-κB to induce acute inflammation and eliminate injury, after which NF-κB activity is terminated by self-limiting mechanisms to safely resolve signaling. However, NF-κB is dysregulated in many inflammatory diseases and leads to persistent inflammation and tissue damage (Figure 4). In the setting of ALI, NF-κB-mediated inflammation and coagulation can each amplify the other, resulting in a vicious cycle of uncontrolled proinflammatory signaling. The central role of NF-κB in disease progression has made it an attractive target for pharmaceutical intervention; small molecule inhibitors have been designed to inhibit NF-κB at multiple steps, ranging from attenuating the ligand/receptor interaction to directly preventing its binding at the promoter [163]. Nevertheless, persistent efforts to target NF-κB in the treatment of critically ill patients have yielded mixed results, likely due to the emerging protective functions of NF-κB (Figure 10A) [33,53,164,165].

Recent studies have identified a role for NF-κB in the resolution of inflammation and injury, complicating its efficacy as a therapeutic target (Figure 10A). NF-κB inhibitors were protective in animal models of ALI when delivered before the peak of injury, whereas NF-κB inhibition during resolution augmented EC barrier injury and delayed tissue repair (Figure 10B) [165,166]. The early expression of a degradation-resistant IκBα mutant (IκBα-m) in the endothelium of septic mice prevented edema formation in the lung and heart, whereas its expression late in sepsis progression increased permeability and EC apoptosis [166]. This distinction between the evolution and resolution phases is important when developing pharmacological therapies, as clinical intervention generally occurs in a patient with a fully established illness or injury (Figure 10A). The dynamics and regulatory mechanisms governing the switch from proinflammatory to anti-inflammatory function of NF-κB throughout injury progression are not well understood and require further investigation; however, some studies have identified key features of NF-κB biology contributing to this differential role. Studies examining the changes in NF-κB signaling during the initiation and resolution of inflammation described shifts in the induced transcriptional programs secondary to the altered composition of the activated NF-κB dimer. Stimulation of the NF-κB pathway during the resolution phase of inflammation/injury induced the transcriptionally inactive p50/p50 homodimer to facilitate healing by repressing the proinflammatory gene transcription characteristic of earlier phases of inflammation/injury [165,167]. In addition to terminating the expression of proinflammatory genes, NF-κB activation during resolution induces protective transcriptional programs [25,26,168,169]. The prosurvival function of NF-κB was shown to defend cells from inflammatory injury and ensure their survival through the recovery phase. Genetic deletion of NF-κB led to massive apoptosis in multiple animal models, causing prolonged and more severe impairment [166,170,171].

In addition to its function during injury, NF-κB is also a key mediator of immune homeostasis. NF-κB has described roles in the maintenance, differentiation, and activation of cells of the innate and adaptive immune system [24,172]. Inflammation is a critical aspect of host defense; however, a careful balance is required to maintain homeostasis [173]. Augmented and persistent inflammation causes tissue injury, as evidenced by numerous NF-κB-driven diseases, whereas abolishing proinflammatory signaling hinders essential host defense systems and provides an opportunity for unimpeded infection (Figure 10B). Trials testing the efficacy of NF-κB inhibitors to treat disease corroborated the risk of eliminating inflammatory signaling. Targeting NF-κB in sepsis patients revealed an initial success in resolving septic injury, but made patients more susceptible to secondary infections [53,174].

**Figure 11:**
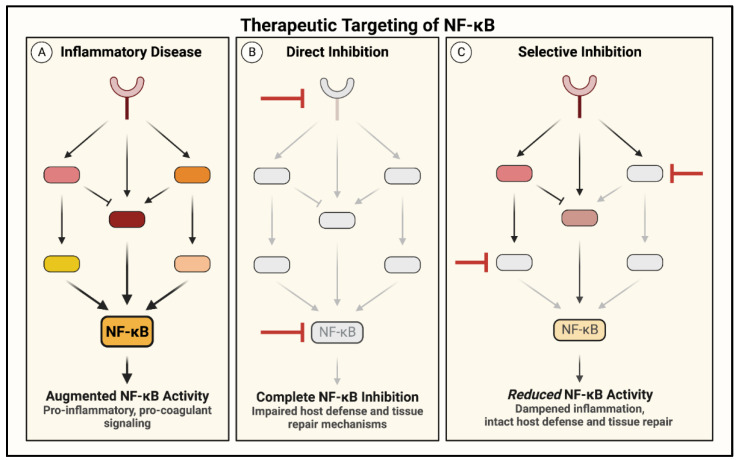
Restraining NF-κB signaling: A promising therapeutic strategy against inflammatory diseases. (**A**) Signaling to NF-κB is augmented in the setting of inflammatory diseases and may be regulated via multiple pathways. (**B**) Direct blockade of the receptor or NF-κB may abolish inflammation, compromising the host defense and tissue repair mechanisms. (**C**) Identification of signaling networks in control of aberrant NF-κB activation and selective targeting of key signaling molecules (alone or in combination) is key to eliminate the detrimental inflammation while maintaining the host defense, tissue repair, and homeostasis functions.

NF-κB integrates numerous upstream signals and translates them into tailored transcriptional responses in a time-, cell-, and stimulus-specific manner, enabling it to play critical roles in injured and healing tissues. The conventional treatment strategy in the setting of inflammatory disease has relied on abolishing NF-κB signaling to limit immune cell recruitment and subsequent organ damage; however, increasing knowledge of its protective roles in injury resolution and immune homeostasis has revealed NF-κB inhibition to be a double-edged sword (Figure 10). Therefore, the key to treating inflammatory diseases such as ALI may lie in “restraining” rather than “abolishing” NF-κB signaling to limit the inflammation while preserving its essential beneficial functions (Figure 11). In this context, restraining or dampening NF-κB selectively in ECs affords the opportunity to: (1) limit the PMN infiltration in the inflamed tissue/lung while preserving the host defense functions of infiltrated PMNs; (2) attenuate the loss of ECs by apoptosis following exposure to oxidants and proinflammatory mediators released from PMNs and other cell types; and (3) support the proliferation of surviving cells to reinstate vascular barrier integrity. Together, these events may facilitate the efficient resolution of inflammatory tissue/lung injury.

## 4. Conclusions

The NF-κB pathway has long been considered a central controller of inflammation, but recent studies have identified additional functions of NF-κB including cell survival and tissue repair [53]. Due to the myriad downstream roles of NF-κB, it has been implicated in both the initiation of inflammation and injury resolution [165,171]. Consistent with this idea, preemptive NF-κB inhibition protects against injury, but inhibition during the resolution phase delays tissue repair [165]. The protective role of NF-κB is not limited to the resolution of injury; it has been shown to be essential for immune homeostasis. Loss of NF-κB signaling in epithelial or parenchymal cells causes the spontaneous onset of severe inflammatory disorders, and in the endothelium, NF-κB maintains homeostatic organization of the vasculature [164,170,175]. Likely for this reason, NF-κB inhibition has yielded disappointing results in the treatment of ALI. This paradoxical regulation of inflammatory injury by NF-κB emphasizes the importance of understanding the complex regulatory mechanisms involved in its pro- and anti-inflammatory functions. A complete blockade of the NF-κB eliminates inflammatory signaling; however, it also hinders beneficial repair functions and compromises the host defense (Figure 11A,B). Therefore, the key to the successful treatment of ALI/ARDS associated with sepsis, pneumonia, and COVID-19 may lie in modulating upstream NF-κB regulatory pathways such that augmented inflammatory signaling is reduced while critical homeostatic and repair mechanisms are maintained. This requires the systematic investigation of the signaling events and their contributions to IKK/NF-κB activation (Figure 11C), which may uncover suitable targets for selectively suppressing the detrimental inflammation without compromising the host defense response.

## Data Availability

Not applicable.
